# Feasibility Analysis of Ultrasound-Guided Placement of Tunneled Hemodialysis Catheters

**DOI:** 10.1016/j.ekir.2023.07.038

**Published:** 2023-08-11

**Authors:** Martin Kächele, Lucas Bettac, Christopher Hofmann, Hannes Herrmann, Amelie Brandt, Bernd Schröppel, Lena Schulte-Kemna

**Affiliations:** 1 Division of Nephrology, Ulm University, Ulm, Germany

**Keywords:** catheter tip position, hemodialysis, interventional nephrology, tunneled central venous catheter, ultrasound-guided placement, vascular access

## Abstract

**Introduction:**

Radiographic fluoroscopy is the current standard for placement of tunneled central venous catheters (CVCs) for hemodialysis. Radiographic fluoroscopy requires structural and personnel infrastructure and exposes the patient to ionizing radiation. Here, we investigate the feasibility of solely ultrasound-guided placement of tunneled central venous dialysis catheters (USCVCs).

**Methods:**

We evaluated prospectively collected single-center data regarding safety and catheter function of 134 consecutive patients who underwent USCVC implantation between 2020 and 2021. We used the inset guidewire to visualize the position of the catheter tip. In the case of inadequate visibility by ultrasound, we used intracardiac electrocardiography (ECG) recording or agitated saline. A total of 1844 catheter days were assessed. The optimal CVC position was defined as being within the upper right atrium (URA) and middle to deep right atrium.

**Results:**

Of the 134 USCVCs, 87% were placed on the right side. The primary success rate for optimal tip position and catheter function was 98%. Of the USCVCs, 97% were placed solely by ultrasound. Regarding positioning, 6% were in the vena cava superior zone, 70% in the URA and 24% in the middle to deep right atrium, resulting in a rate of 94% with optimal positioning. Effective blood flow averaged 292 ± 39 ml/min. There were no immediate procedure-associated complications.

**Conclusion:**

Placement of CVC for hemodialysis solely by ultrasound is an effective alternative to fluoroscopy-assisted placement.

For end-stage kidney disease, renal replacement is needed to sustain and maintain life. The arteriovenous fistula is the preferred access for most patients opting for hemodialysis. The implantation of tunneled central catheters is often required for urgent and temporary vascular access. Placement of tunneled-cuffed catheters is preferably performed using fluoroscopy.^1^ In addition to exposing the health care team and patients to potentially harmful radiation, fluoroscopy requires appropriate equipment and infrastructure, increasing the costs of this procedure.^2^^,^^3^

Proper CVC placement is critical to ensuring optimal function and avoiding complications of poor function or repeat interventions. For nontunneled CVC, limited data are available regarding the preferred method to ensure optimal positioning. Guidelines suggest using fluoroscopy or ultrasound for the insertion to reduce complications.^4^ To verify the correct position of the catheter tip, fluoroscopy is commonly used.^1^

Other techniques, however, exist to guide the placement of catheters without fluoroscopy. For nontunneled CVC, ECG-assisted implantation in intensive care units and during surgery is superior to a solely anatomically oriented implantation by landmark technique.^5^^,^^6^ The ECG-assisted method is used to facilitate the placement of tunneled CVC for dialysis and can still be used in patients with atrial fibrillation.^7^^,^^8^ During COVID era, successful and safe bedside placement of tunneled CVC using anatomic landmarks has been reported.^9^ Recently, for tunneled dialysis CVC, a technique using agitated saline for localization of the catheter tip near the atrium has been described.^10^^,^^11^

Nonetheless, all these techniques provide indirect or limited information about right atrial structures (e.g., tricuspid valve, filling state) and the position of the catheter tip relative to them.

Here, we describe a novel implantation technique for tunneled-cuffed hemodialysis CVC using only ultrasound for tip positioning.

## Methods

### Patient Population and Study Design

A total of 134 consecutive patients who were scheduled for a tunneled-cuffed dialysis CVC between January 2020 and December 2021 consented to USCVC placement. We prospectively collected data on the cause of kidney disease, comorbidities, previous dialysis access, catheter performance, and complications.

The success rate of the catheter tip position after USCVC placement was compared to 50 historical controls who had received fluoroscopy-guided placement from January 2018 until December 2019. The study was approved by the local institutional review board.

### Ultrasound-Guided Catheter Placement

USCVC placement was performed under continuous circulatory monitoring. All patients received local anesthesia and opiate-based analgesia and sedation as needed.

After disinfection and sterile draping, the target vessel was punctured with ultrasound guidance and control using an 18 G puncture needle and pushing forward a 0.97 mm nitinol-coated J-guidewire. As standard approach, we used the jugular vein. Subclavian vein access was only used as a last resort. Correct placement of the wire toward the right atrium was verified by a subcostal cardiac view. The ultrasound system (Mindray TE 5, Mindray Medical, Darmstadt, Germany) with a 5 MHz convex probe was optimized for the visualization of the heart and guide wires by adjusting the presets (reduction of dynamic range and width angle).

After verifying that the wire was correctly placed, we proceeded by preparing a subcutaneous pocket at the jugular turnover point and dilation of the access channel. A double-lumen cuffed silicone dialysis catheter (Joline, Hechingen, Germany), 6.1 × 3.5 mm, was inserted while using ultrasound to visualize the right atrium.

**Figure 1:**
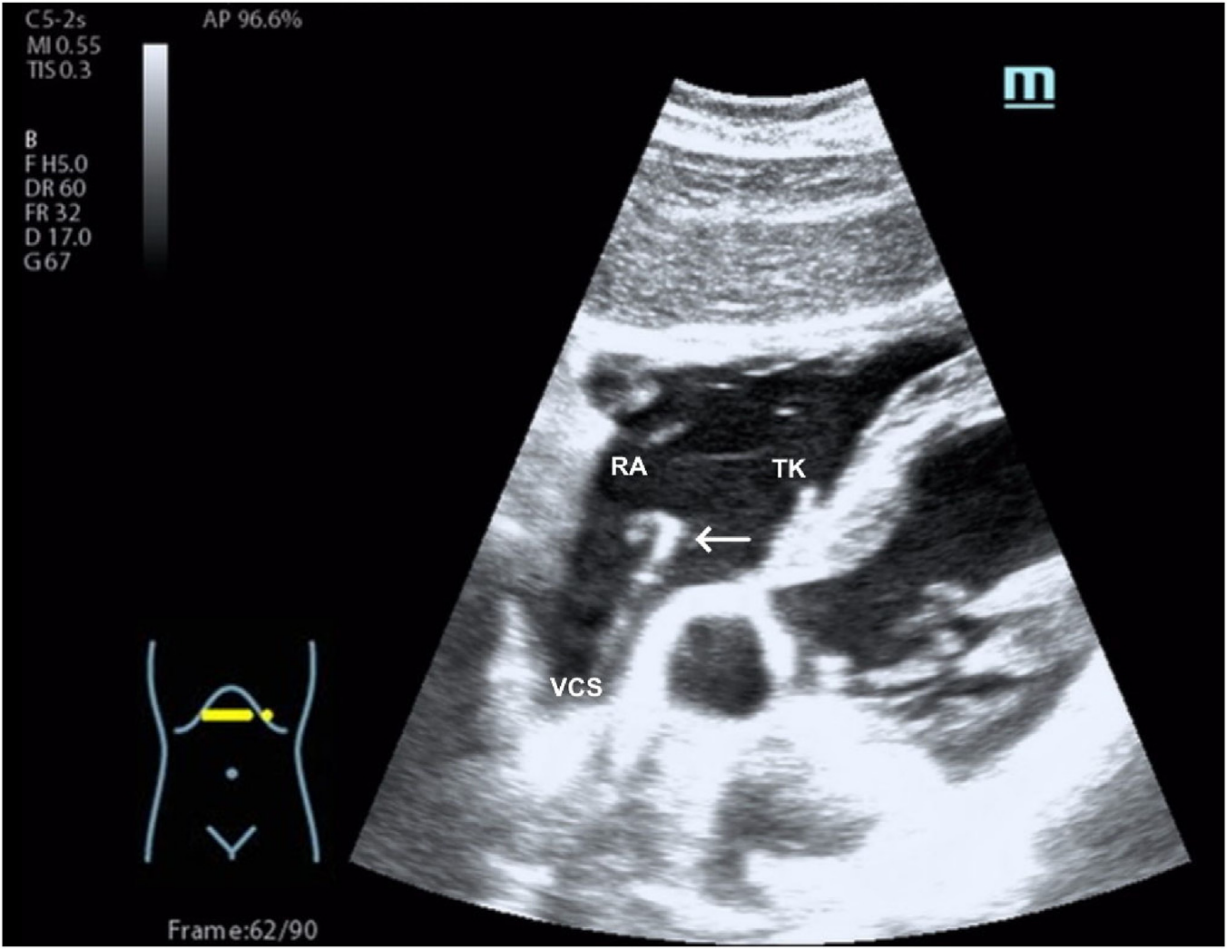
Ultrasonographic visualization of the right atrium for catheter positioning. Ultrasonographic visualization of the right atrium for catheter positioning. Catheter and guidewire are aligned with the J protruding from the catheter tip for a better contrasting of the catheter tip in the middle right atrium. RA, right atrium; TK, tricuspid arteriovenous valve; VCS, superior vena cava; Arrow, catheter tip with J-wire.

For positioning, first the catheter and J-guidewire were aligned with the J shortly protruding from the catheter tip to get a better contrast with the catheter. Precise alignment of catheter and guide wire tip was ensured by the resistance of the J-end at the position of catheter tip during wire retraction and the externally visible markings of the wire. This visually enhanced catheter was then placed with its tip between the cavoatrial junction and the middle right atrium. The main focus was ensuring that the catheter tip was free of contact with the tricuspid valve or the atrial floor, and that the arterial lumen was facing the mediastinum (Figure 1, Supplementary Video S1).

In the case of inadequate or absent visualization by ultrasound alone, we first performed an intracardiac ECG lead (Vygocard, Vygon, Aachen, Germany; Philips IntelliVue MX400) via the guide wire. In the case of insufficient signal, agitated saline was injected through the catheter.^10^ Retrograde subcutaneous tunneling was performed using a trocar. The procedure was carried out by 2 team members.

All patients received a postprocedural chest X-ray to document the position of the catheter and exclude complications (e.g., pneumothorax). Catheter function during dialysis as well as periprocedural and postprocedural complications were documented until hospital discharge or transfer to an outpatient dialysis center.

### Fluoroscopic-Guided Catheter Placement (Historical Control)

As postoperative chest X-ray imaging was not available for all patients, only patients with admission and postoperative dialysis treatment were included in the analysis. Due to retrospective data collection, procedure-associated and postoperative complications were not consistently available.

### Study Outcome

The primary composite outcome was the primary success rate, a combination of catheter function (catheter functionality or fitness for hemodialysis treatment, described by the maximum documented effective mean blood flow rate during the observation period) and position (no need for replacement or correction after postinterventional X-ray). The position of the catheter tip in the chest radiograph was categorized into 3 areas. The zone vena cava superior refers to the area above the cavoatrial junction, the zone URA refers to the segment between the cavoatrial junction and the middle right atrium, the zone right atrium refers to the middle to deep right atrium (Supplementary Figure S1).

Secondary outcomes were procedure-related complications, catheter-associated infections and complications by the end of the observation period.

### Statistical Analyses

Normally distributed variables are expressed as mean values with standard deviation. Qualitative variables were expressed as percentages. Statistical differences between groups were determined by Wilcoxon rank sum (for age and body mass index), Fischer’s exact *t* test (for body size and weight) or Pearson’s chi-square (for sex, side of access, catheter position).

## Results

### Population

A total of 134 patients with a USCVC placement were included in the analysis. The mean age was 64.5 ± 16.7 years; baseline characteristics are shown in Table 1.

**Table 1:** Baseline characteristics

Characteristics	*N* (%)
Total	134
Demographics and comorbidities	
Age (yrs) ± SD	64.5 ± 16.7
	IQR 57–77.8
Male	78 (59)
BMI (kg/m^2^) ± SD	27.6 ± 7.7
Diabetes	50 (37)
Hypertension	95 (70.8)
Chronic heart failure	52 (38.8)
Cardiovascular disease	40 (30)
Lung disease	26 (19.4)
Atrial fibrillation	31 (23)
Pacemaker/port system	14 (10.4)
Immunosuppressive therapy	29 (21.6)
Anti-platelet therapy	41 (30.6)
Anticoagulation	12 (8.9)
Dialysis modality	
Urgent start of hemodialysis	110 (82)
Via tunneled catheter	40 (29.8)
Switch from PD	10 (7.5)
Arteriovenous fistula failure	2 (1.4)
Previous dialysis catheters	
0	46 (34.3)
1	76 (56.7)
≥2	12 (9.0)
Laboratory values	
Hemoglobin [g/dl]	9.05 ± 1.5
Thrombocytes [/nl]	205 ± 95
Serum creatinine [μmol/l]	514 ± 230
Serum albumin [g/l]	33.5 ± 10.1
Urea [mmol/l]	17.8 ± 12.8
INR	1.1± 0.2
PTT (sec)	31.9 ± 7.5

BMI, body mass index; IQR, interquartile range; INR, international normalized ratio; PD, peritoneal dialysis; PTT, partial thromboplastin time.

Of the 134 cases analyzed, 110 patients had an unplanned urgent hemodialysis start. Reflecting common practice, these patients are commonly dialyzed by a nontunneled catheter and later converted to a tunneled catheter; however, 40 of 110 urgent-start patients were started on dialysis by a tunneled catheter without prior nontunneled access.

### Catheter Position

The USCVC placement was feasible in all cases (*n* = 134). One case was excluded from the analysis because of a missing postinterventional X-ray. In 130 cases, positioning of the catheter tip was performed solely by ultrasound. In 4 cases, due to inadequate quality of the ultrasound image to assess the CVC position, intracardiac ECG recording (*n* = 2) and agitated saline (*n* = 22) were used. In 1 of these 4 cases, using agitated saline for confirmation, the final radiograph showed the catheter tip in the superior vena cava.

### Catheter Function

All patients received at least 1 hemodialysis treatment at our facility after the procedure. Primary dysfunction was reported for 1 catheter due to a kink at the jugular envelope point. After correction of the kink, the catheter was functional.

The medium effective blood flow was 292 ± 39 ml/min. Limited follow-up data existed for a relevant proportion of USCVC. As per local protocol, a reduced blood flow (200–250 ml/min) in the first 2 to 3 hemodialysis treatments was applied. Higher blood flow was briefly applied for functional control and not always documented. To investigate whether a sustained lower blood flow was also applied in routine use (as a possible indicator for malfunction), we excluded patients with dialysis initiation (the period up to 4 days after the first dialysis). In 9 cases, a blood flow <250 ml/min was used during this initial period; Excluding these cases, the mean blood flow was 297 ± 35 ml/min. In 1 case, a blood flow of 235 ml/min was applied on day 5 of dialysis (without a documented cause).

The composite primary end point for position and function was 98% (Table 2).

**Table 2:** Primary outcome variables

Primary success rate	*N* (%)
Function and position	131 (97.7)
Placement solely be ultrasound	130 (97)
Additional use of intracardiac electrocardiography	2 (1.5)
Additional use of agitated saline	2 (1.5)
Primary dysfunction, malposition, revision	
Wrong position (intravascular kinking)	1 (0.7)
Kinking at jugular turnover point	1 (0.7)
Blood flow < 250 ml/min at day 5	1 (0.7)
Mean blood flow rate	292 ± 39 ml/min

### Complications

The frequencies of complications are listed in Table 3. Puncture of the carotid artery occurred in 5.9% (*n* = 8), all of which were without sequelae. Two patients had a decrease in hemoglobin of more than 2 g/dl. Severe bleeding requiring blood transfusion did not occur. Two patients died within 48 hours after the procedure. In 1 case, it resulted from sedation-induced aspiration pneumonia with respiratory failure; in the second case, from sudden cardiac arrest that was most likely the result of pulmonary embolism. No direct causal relationship with the type of intervention (perforation, hemorrhage, tamponade, or pulmonary injury) could be found. Catheter-related infections were not reported during the 1844 catheter days during the observation period. In 1 patient, the catheter had been removed due to suspected infection, which was not confirmed on subsequent cultures.

**Table 3:** Complications

Complications	*N* (%)
Mortality	4 (2.9)
Death <48 hours	2 (1.5)
- Pulmonary embolism	1
- Aspiration pneumonia	1
Hemoglobin fall >2 g/dl, transfusion	2 (1.5)
Arterial puncture	8 (5.9)
Catheter withdrawal	3 (2.2)
- Accidental removal by patient	1
- Suspected infection (unconfirmed)	1
- No longer required	1
Catheter related infection	0 (0)
Thrombolysis	1 (0.7)

### Cather Tip Position of USCVC Compared to Fluoroscopy

**Figure 2:**
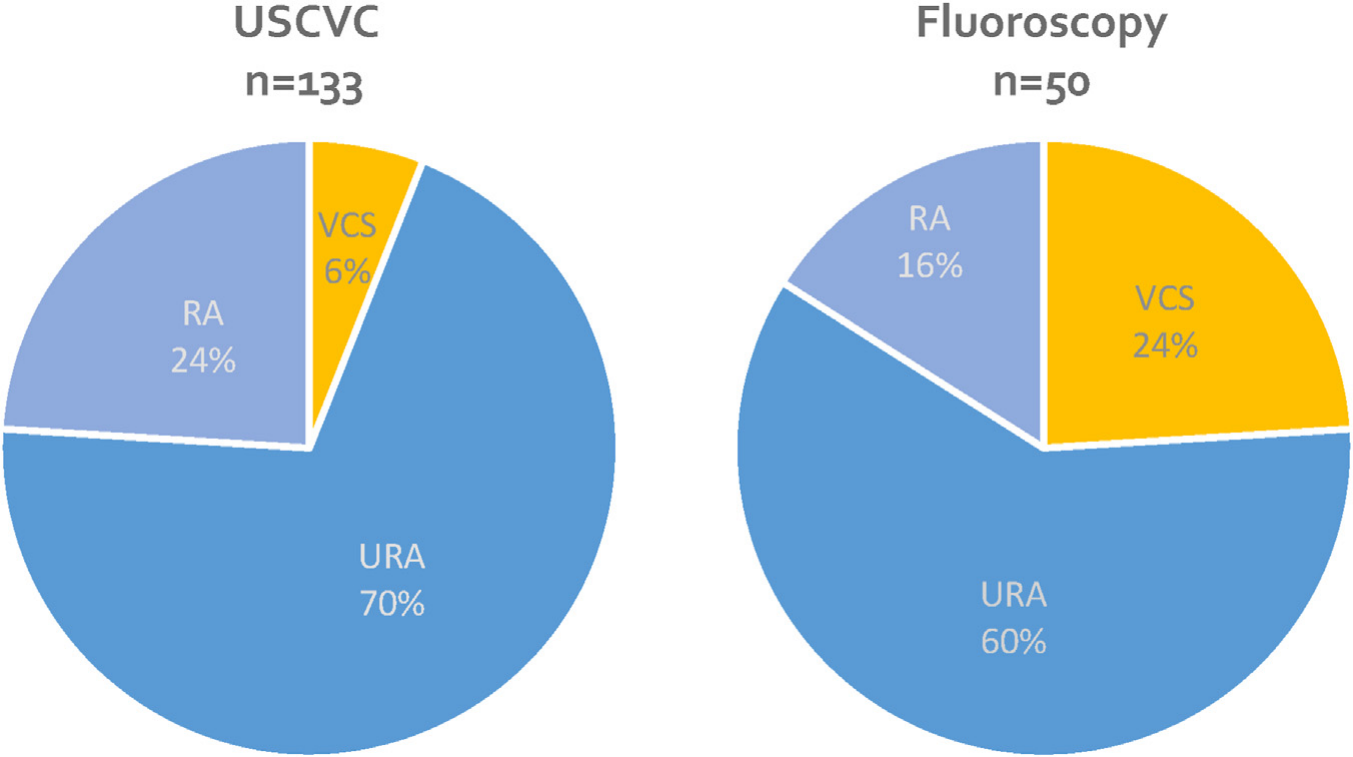
Catheter tip position after ultrasound-guided (*N* = 133) and fluoroscopy-guided catheter placement, *P* = 0.0005, Pearson’s chi-square. RA, right atrium; VCS, vena cava superior; URA, upper right atrium; USCVC, ultrasound-guided central venous catheter.

We compared the tip position of USCVC with a historical cohort placed under fluoroscopy (Table 4). In this regard, 70% of USCVC were in the URA (60% by fluoroscopy), 6% in the superior vena cava (24% by fluoroscopy), 24% in the right atrium (16% by fluoroscopy). Therefore, USCVC had a higher proportion of tips in the target area (URA and middle to deep right atrium) (Figure 2).

**Table 4:** Procedure related variables

Characteristics	USCVC	Historical controls	*P*
	*n* (%)	*n* (%)	
Total number (*n*)	134	50	
Age (yrs)	64.5 ± 16.7	64.7 ± 17.5	0.74^a^
	IQR 57–78	IQR 57–77	
Male	78 (59)	30 (60)	0.87^b^
BMI (kg/m^2^)	27.6 ± 7.7	30.2 ± 8.9	0.04^a^
Catheter location			
Right	117 (87.3)	46 (92)	0.37^b^
Left	17 (12.7)	4 (8)	
Jugular vein	130 (97)	50 (100)	
Subclavian vein (right)	4 (3)	0 (0)	
Duration of surgery (min)	27 ± 12	NA	

AV, arteriovenous; BMI, body mass index; NA, not available; PD, peritoneal dialysis; USCVC, ultrasound-guided central venous catheter placement.

a Wilcoxon rank sum test.

b Pearson’s chi-square test.

A comparison of the blood flow rate of USCVC versus fluoroscopy was not performed due to the high proportion of single lumen catheters used in the fluoroscopy group.

## Discussion

Here, we report a single-center experience with ultrasound-assisted placement of tunneled cuffed hemodialysis catheters. The procedure performed on 134 consecutive patients was safe and efficient. The technique was feasible in all cases and resulted in a 97.7% success rate for the composite outcome of catheter position and function. The complication rate in our cohort was low and comparable to previous studies with a population consisting predominantly of patients with unplanned or emergency dialysis initiation.^2^^,^^12^^,^^13^ A retrospective analysis reported a procedural success rate for function and position of 98% using fluoroscopy and 92% using landmark technique.^2^ A combined primary success rate of 93% (6 malpositions and 3 malfunctions) was found in a retrospective study of 142 patients receiving a tunneled dialysis catheter by intracardiac ECG.^8^

Complication rate was generally low; however, studies differ regarding the population (elective vs. acute start) and possibly underestimate the true rate due to their retrospective design. Of note, one of the few prospective studies in hemodialysis patients reporting mortality excluded 2.3% of patients because of death between catheter implantation and first dialysis.^14^ The incidence of carotid puncture in our study was with 5.8% higher than reported in a Cochrane review 2.6%, ranging from 1.7% to 4.1%.^15^

Ultrasound-assisted placement ensures direct visibility of the catheter tip in relation to the anatomical structures. This will avoid excessively deep positioning in the inferior vena cava or positioning in proximity to the tricuspid valve. Furthermore, it is possible to find the best position within the right atrium even in the case of a low atrial filling. Of note, reliable sonographic visualization of the catheter is only possible with the guide wire in place. The J-tip, placed directly at the distal end of the catheter is an easily identifiable landmark that allows safe placement of the catheter tip.

Although a subcostal view is sufficient in the vast majority of cases, proficiency in ultrasound technique and imaging quality are critically important.

Although there is very little data available on the use of solely ultrasound-guided placement of tunneled dialysis catheters, this technique was recently described in a large cohort of nontunneled hemodialysis catheters.^16^ Here, the guidewire was located either in the atrium or in the inferior vena cava, as a marker of a correct orientation. The authors describe a high success rate. However, different to our method, the wire was not used as a guide to place the catheter tip in the correct atrial position.^16^

In our study, we were not able to clearly identify risk factors which would influence success rate or procedure time, such as body mass index, insertion site, or number of previous catheter insertions.

We were unable to clearly visualize the catheter tip position in only 3% of cases. Additional use of intracardiac ECG recording or agitated saline injection ensured correct positioning in these cases.

Indirect methods such as ECG or agitated saline injection have limitations. In 1 case, the rapid flooding of infused bubbles was misinterpreted as the correct position, but later showed a short-distance turnover of the tip into the superior vena cava in the post-procedural X-ray.

Visibility of bubbles in the right atrium within 2 seconds after injection has been postulated to be a sufficient criterion for a correct position. Visualization of the catheter tip position using agitated bubbled saline has been used in a cohort of 57 patients with excellent results.^10^ For tunneled dialysis catheters, a larger study in 142 patients from South Korea showed that the ECG-guided system assured good catheter positioning and function.^8^

Nevertheless, others found that in a cohort of nontunneled catheters, the central position of the catheter tip might be overestimated in the case of left-sided insertion during ECG-supported catheter placement.^6^ In these cases, the catheter tip is positioned too far cranially in the superior vena cava, which could be disadvantageous for the function of hemodialysis catheters.

The position of the tip in the superior vena cava was associated with a lower catheter survival after 6 months.^14^ It is notable, that our ultrasound technique provided a high likelihood that catheters end between the cavoatrial junction or in the middle to deep atrium. This position is consistent with current Kidney Disease Outcomes Quality Initiative recommendations^1^ and is preferred due to better function.^17^ In addition, it is possible to adapt the catheter placement to individual anatomical characteristics. On X-ray, 6% of catheter tips ended in the superior vena cava. Studies comparing fluoroscopic landmarks with direct visualization by transesophageal echocardiography showed that neither radiographic nor vertebral landmarks can reliably determine the correct cavoatrial transition zone.^18^

In principle, the right side should be preferred for atrial catheterization, especially if the creation of an arteriovenous fistula is planned in the future. In some cases, it was necessary to deviate from this approach. It is conceivable that without fluoroscopy, the risk of vascular complications increases with left-sided placement, though others could not confirm this.^2^

In our cohort, the small number of left-sided catheters (*n* = 17) did not allow any statistically valid conclusion. The left-sided placement can be challenging due to a more difficult tip visualization. Despite this, we did not observe a longer surgery time, a higher failure rate, or an increase in bleeding or other complications. The catheter we used does not require a rigid split-stealth sheath due to the retrograde placement technique. The rigid dilator was advanced only as far as it entered the vein in each case to avoid perforation of deeper sections. The catheter material in our case is silicone, in order to avoid vascular complications due to high rigidity.

The main limitation of our study is the single center and single arm design. However, all USCVC placed during this period were evaluated, avoiding a selection bias. Our historical controls used different types of catheters, a different placement technique (with and without split sheath), and the data were analyzed retrospectively. Therefore, it was not possible to make any comparative statements about the functionality or complication rate.

## Conclusion

Ultrasound-guided placement of tunneled cuffed hemodialysis catheters is an effective alternative to fluoroscopy.

## Disclosure

MK participates as principal investigator in a post-marketing clinical follow up study for dialysis catheters sponsored by Joline (Hechingen, Germany). All the other authors declared no competing interests.

## Supplementary Material

Supplementary Video S1 (video)

**Video S1.** Sonographic placement of tunneled hemodialysis catheters.

**Figure S1.** Definition of tip position zones in chest x-ray.
