# Supplementary material for: Feasibility Analysis of Ultrasound-Guided Placement of Tunneled Hemodialysis Catheters
Source: Kidney Int Rep. 2023 Aug 11;8(10):2001–7. doi: 10.1016/j.ekir.2023.07.038 (PMC10577359; doi:10.1016/j.ekir.2023.07.038)
Supplement: Supplementary File (PDF) [file mmc1.pdf]

Supplementary Material

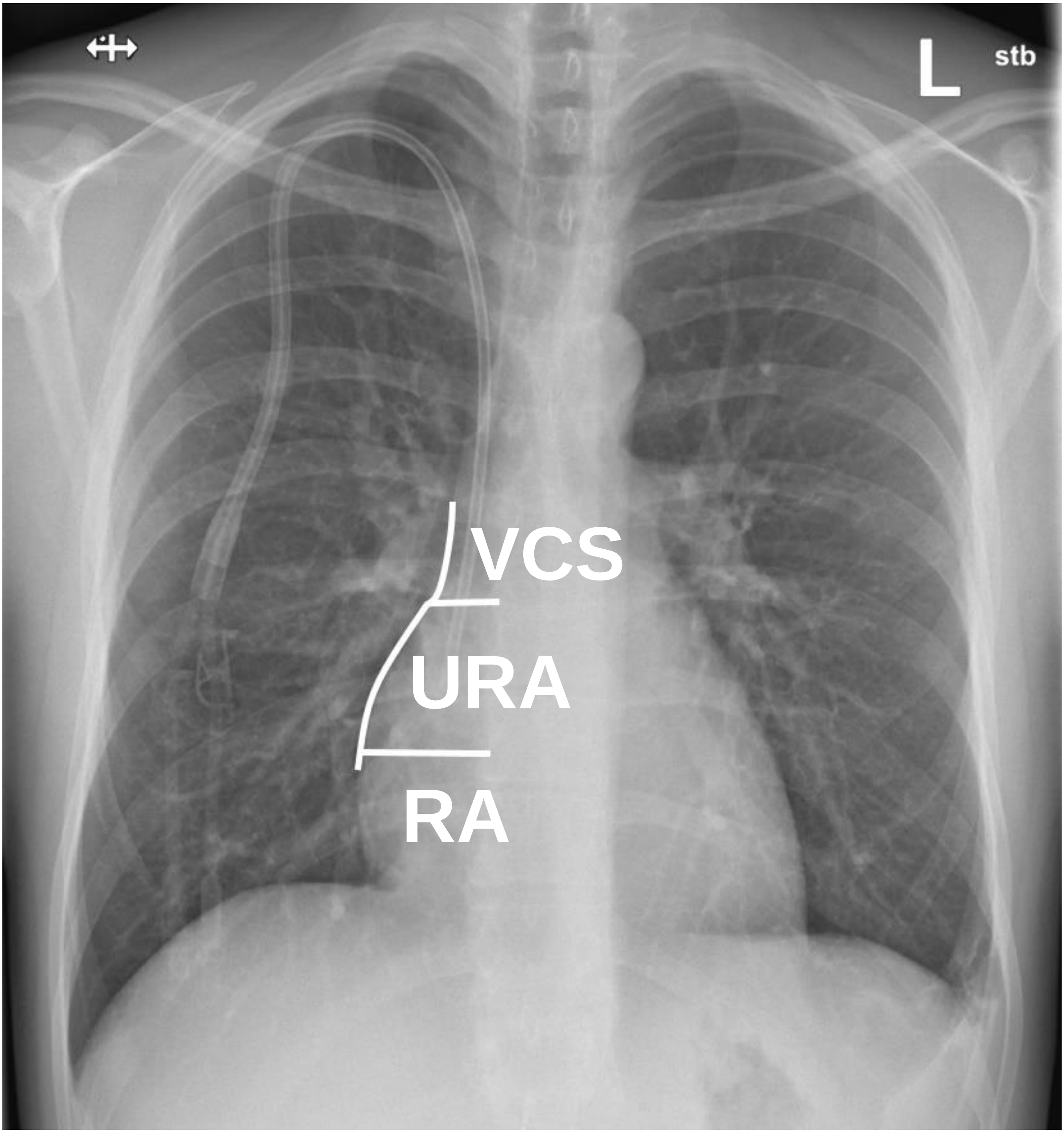

Figure S1: Definition of tip position zones in chest x-ray

RA: right atrium, VCS: vena cava superior, URA: upper right atrium
